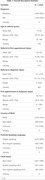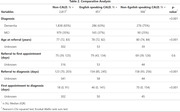# Does cultural and linguistic diversity impact timeliness of dementia diagnosis? Findings from the Australian Dementia Network Registry

**DOI:** 10.1002/alz.089766

**Published:** 2025-01-09

**Authors:** Susannah Ahern, Xiaoping Lin, Mohammad Amin Honardoost, Kasey Wallis, Henry Brodaty, Stephanie Alison Ward, ADNetT Registry Steering Committee

**Affiliations:** ^1^ Monash University, Melbourne, VIC Australia; ^2^ University of New South Wales, Sydney, NSW Australia

## Abstract

**Background:**

The Australian Dementia Network (ADNeT) is a collaboration of dementia researchers and clinicians established in 2018. It includes a clinical quality registry that reports on diagnosis and early management of people with dementia or Mild Cognitive Impairment (MCI) across public, private, metropolitan and rural settings. Australia is multicultural and the registry collects information regarding cultural and linguistic diversity (CALD). The aim of this study was to determine if CALD status was associated with time to dementia diagnosis.

**Methods:**

ADNeT registry data from March 2020 – Sept 2023 were analysed. Primary endpoint was time to diagnosis for three groups based on CALD status and preferred spoken language: non‐CALD; English‐speaking CALD; and non‐English‐speaking CALD. Descriptive statistics, univariate and multivariate regression analysis were undertaken for five outcome measures: (1) time from referral to diagnosis (2) time from referral to first appointment, (3) time from first appointment to diagnosis (4) age at referral, and (5) diagnostic outcome (dementia vs MCI).

**Results:**

Of 3,634 participants, 2,817 (78%) were non‐CALD; 451 (12%) were English‐speaking CALD and 366 (10%) were non‐English speaking CALD. Overall median times were 131 days from referral to diagnosis, 75 days from referral to first appointment, and 28 days from first appointment to diagnosis. Both English and non‐English‐speaking CALD background were associated with longer time from referral to diagnosis (154/158 days vs 123 days for non‐CALD), and longer time from first appointment to diagnosis (46/70 days vs 18 days) (p<0.001), remaining significant after adjusting for demographic/health factors. There was no association between CALD and time from referral to first appointment. Non‐English‐speaking CALD background was also associated with older age at referral (80 years vs 78/77 years) and a higher likelihood of dementia diagnosis (75% vs 63/65%), although these differences disappeared after adjustment.

**Conclusion:**

This large national study shows that cultural and linguistic diversity is associated with delayed diagnosis of dementia, with further research required regarding underlying causes. This has important implications for equity of care as it may preclude people from CALD backgrounds accessing novel disease modifying treatments. People from CALD backgrounds may specifically benefit from dementia diagnostic biomarkers to facilitate earlier diagnosis.